# The Mitochondrial Architecture of Perinatal Mental Disorders

**DOI:** 10.1192/j.eurpsy.2026.11946

**Published:** 2026-07-31

**Authors:** B. Larisa

**Affiliations:** Department of Mental Health, Medical Psychology and Psychotherapy, Nicolae Testemițanu State University of Medicine and Pharmacy, Chisinau, Moldova, Republic of

## Abstract

**Introduction:**

Perinatal affective and psychotic disorders arise during rapid endocrine and immunometabolic remodeling. Evidence supports a stress–mitochondria–inflammation axis linking peripheral and central signals via cell-free mitochondrial DNA (cf-mtDNA), cytokines, and extracellular vesicles (including exosomes).

**Objectives:**

To integrate mitochondrial, immune, and lipid mechanisms into a translational framework and propose a biomarker panel for dynamic risk assessment across pregnancy and early postpartum (<=12 weeks).

**Methods:**

Narrative synthesis of PubMed/MEDLINE, Scopus, and Web of Science (2019–September 2025). Included studies investigated perinatal mental states (pregnancy to <=12 weeks postpartum) with depressive and/or psychotic phenotypes and reported mitochondrial indices (reactive oxygen species, OXPHOS, cf-mtDNA), cytokines/extracellular vesicles, placental ferroptosis markers (TFRC, ACSL4, ALOX5, GPX4), lipid peroxidation products (MDA), or modulators (omega-3 DHA; nicotinamide/NAD+). Exclusions: editorials, single-case reports without biomarkers, studies outside the peripartum window, and unavailable English full text. A formal risk-of-bias assessment was not undertaken given the narrative scope.

**Results:**

(1) Mitochondrial stress and released mtDNA engage innate sensors (TLR9, NLRP3, cGAS–STING), promoting IL-1beta, IL-6, and TNF-alpha signaling. (2) Pregnancy-associated psychosis correlates with a placental ferroptosis profile—upregulation of TFRC/ACSL4/ALOX5, downregulation of GPX4, and increased lipid peroxidation—potentially linking iron-driven peroxidation to adverse obstetric and psychiatric outcomes. (3) Preclinical gestational-stress models show reduced medial prefrontal cortex (mPFC) complex I–dependent OXPHOS and impaired maternal behaviors; nicotinamide, which is a NAD+ precursor, preserves respiration and behavior in these models. (4) A micro-panel for longitudinal risk and prevention monitoring is proposed: cf-mtDNA (± EV-associated), IL-6 (± hs-CRP), glutathione redox ratio, lactate/pyruvate ratio, and kynurenine/tryptophan ratio; optionally immune-cell oxygen consumption and extracellular acidification rates.

**Conclusions:**

Perinatal mental disorders share a mitochondrial–inflammatory architecture with placental and lipid peroxidation components. Composite biomarkers warrant prospective validation in well-phenotyped cohorts, and standardized preanalytical protocols for cf- and EV-associated mtDNA are required before clinical translation. NAD+ precursors and omega-3 strategies are promising but require randomized evaluation.

**Keywords:**

perinatal depression; postpartum psychosis; mitochondria; cf-mtDNA; extracellular vesicles; ferroptosis.

**Disclosure of Interest:**

None Declared

